# Avoiding an impending collision in international conservation

**DOI:** 10.1111/cobi.14450

**Published:** 2025-02-10

**Authors:** Lovemore Sibanda, Amy Dickman, Courtney Hughes, Jessica Tacey, Emily Madsen, Lessah Mandoloma, Moreangels M. Mbizah, Yolanda Mutinhima, Betty Rono, Salum Kulunge, David Kimaili, Trisha Bhujle, David W. Macdonald, Darragh Hare

**Affiliations:** ^1^ Department of Biology Oxford University Oxford UK; ^2^ Wildlife Conservation Research Unit, The Recanati‐Kaplan Centre, Department of Biology Oxford University Tubney UK; ^3^ Cheetah Conservation Project Zimbabwe Dete Zimbabwe; ^4^ Forestry & Parks Government of Alberta Edmonton Alberta Canada; ^5^ Department of Environmental Science and Natural Resources Management Lilongwe University of Agriculture and Natural Resources (LUANAR) Lilongwe Malawi; ^6^ Wildlife Conservation Action Harare Zimbabwe; ^7^ Department of Wildlife, Ecology and Conservation Chinhoyi University of Technology Chinhoyi Zimbabwe; ^8^ Department of Zoology and Entomology Rhodes University Grahamstown South Africa; ^9^ NARE Egerton University Njoro Kenya; ^10^ Tanzania Wildlife Management Authority Morogoro Tanzania; ^11^ Department of Wildlife Management Sokoine University of Agriculture Morogoro Tanzania; ^12^ Department of Sociology and Anthropology South‐Eastern Kenya University Kitui Kenya; ^13^ Department of Natural Resources and the Environment Cornell University Ithaca New York USA

**Keywords:** 30×30, inclusive conservation, IPLCS, Protected and Conserved Areas, Protected-Area governance

There are long‐standing tensions between 2 major movements in international conservation: one emphasizes increasing the area set aside for conservation and the other emphasizes an inclusive, people‐centered approach to conservation. The degree to which these movements harmonize or contradict depends largely on how decision makers balance strictly protected areas (PAs) with flexible other effective area‐based conservation measures (OECMs).

The United Nations Convention on Biological Diversity's Kunming–Montreal Global Biodiversity Framework (GBF) embodies elements of both movements. Target 3 (30×30) sets ambitious targets to protect 30% of inland water, land, and marine ecosystems by 2030 through a combination of PAs and OECMs while respecting the rights of Indigenous People and local communities (IPLCs) (CBD, 2022). The final wording is

Ensure and enable that by 2030 at least 30 per cent of terrestrial, inland water, and of coastal and marine areas, especially areas of particular importance for biodiversity and ecosystem functions and services, are effectively conserved and managed through ecologically representative, well‐connected and equitably governed systems of protected areas and other effective area‐based conservation measures, recognizing indigenous and traditional territories where applicable, and integrated into wider landscapes, seascapes and the ocean, while ensuring that any sustainable use, where appropriate in such areas, is fully consistent with conservation outcomes, recognizing and respecting the rights of indigenous peoples and local communities, including over their traditional territories.

Over 190 countries have ratified the CBD and committed to the GBF (WWF & IUCN WCPA, 2023), but opinions regarding the motivations for and implications of 30×30 are mixed. Proponents are optimistic that it will deliver substantial positive impacts for biodiversity (Waldron et al., 2020; Wolff et al., 2023), whereas critics argue that it risks prioritizing the goals and interests of people living far from biodiversity‐rich areas over those of marginalized IPLCs (Green Economy Coalition, 2021; Rudd et al., 2021).

The discourse illuminates tensions between traditional area‐based conservation via formal PAs and calls for more inclusive, people‐centered approaches (Bakarr, 2023; IUCN Africa Protected Areas Congress, 2022). The people‐centered approach, or inclusive conservation, contends that conservation has traditionally excluded IPLCs from PAs, for example, by preventing sustainable access to and use of wildlife resources (Lo & Jang, 2022). Therefore, the people‐centered approach seeks to simultaneously conserve biodiversity and improve outcomes for IPLCs who have been or continue to be marginalized by area‐based conservation (Raymond et al., 2022).

As conservation researchers and practitioners working in multiple landscapes, we have seen how global conservation movements influence decisions that affect PAs and OECM management and, therefore, IPLCs. We appreciate that 30×30 recognizes OECMs and formal PAs (Cook, 2024), but we are concerned that achieving inclusive conservation under 30×30 will depend on how decision makers define and interpret PAs and OECMs. If new PAs and OECMs are designated following traditional exclusionary methods or if PA and OECM management strategies are defined without fully incorporating the rights, values, needs, and concerns of IPLCs, efforts to deliver 30×30 might unintentionally reproduce historical inequalities and reinforce power imbalances associated with colonial forms of conservation (Rudd et al., 2021; Willow, 2016).

We therefore see an impending collision at the heart of 30×30. To avoid this collision, the voices of IPLCs must be included in ongoing debates and decision‐making about how and where to conserve biodiversity (Sandbrook et al., 2023). This could involve establishing, supporting, and expanding comanagement models to ensure conservation measures are aligned with IPLCs’ knowledge and needs (Rocha et al., 2017). One example is Yaigojé Apaporis National Park in Colombia, which was created at the request of Indigenous Peoples and managed in collaboration with them (Huaiquimilla‐Guerrero et al., 2023).

Conservation efforts (including PAs and OECMs) can better reflect local interests by implementing governance models that decentralize power dynamics (Cebrián‐Piqueras et al., 2023). Without meaningful collaborative decision‐making with IPLCs, local resentment and opposition to conservation are likely and could result in neither biodiversity conservation nor social justice (Bennett et al., 2019; Sandbrook et al., 2023). This risk challenges conservation scientists, practitioners, and decision makers to better define *effective conservation* and evaluate effectiveness over time (Lee & Abdullah, 2019).

To meet commitments under target 3, national decision makers must find a balance between strict PAs and more flexible OECMs. They must also create a delicate balance among the needs, interests, and concerns of people living in high‐biodiversity areas and those living elsewhere in a country (Dawson et al., 2024). Doing so could help ensure that 30×30 produces practical area‐based solutions to the worldwide biodiversity crisis without further marginalizing IPLCs (IUCN Africa Protected Areas Congress, 2022). This should also involve recognizing and respecting IPLCs’ expertise and ability to effectively conserve biodiversity outside formal PAs (ICCA Consortium, 2021) and respecting IPLCs’ agency via shared leadership, as opposed to treating IPLCs as convenient partners in delivering a vision for area‐based conservation defined by others (Busck‐Lumholt et al., 2024; Dawson et al., 2024).

Realistically, the costs of delivering 30×30 will largely fall on IPLCs living near biodiversity‐rich areas, even though more powerful—often distant—actors in the Global North champion the target (Earsom, 2023). Therefore, to avoid 30×30 reproducing colonial inequalities, such as displacing and further marginalizing IPLCs, politicians, businesses, nongovernmental organizations, and funding organizations could provide more direct financial support to IPLCs (Sangha, 2020). Such financial support could empower IPLCs to manage and preserve their natural resources effectively, according to their own cultures and values (Jeanty, 2021).

Although IPLCs receive financial support from multiple sources (approximately US$270 million per year over the last 10 years [United Nations Environment Programme, 2021]), there remains a huge gap between available funding and actual needs on the ground (Larson et al., 2022). Ensuring that IPLC conservation efforts receive adequate funding and that IPLCs are directly involved in decision‐making could foster a more equitable and sustainable approach to global biodiversity conservation (Busck‐Lumholt et al., 2024; Milner‐Gulland, 2024).

The potential collision between expanding area‐based conservation measures and inclusive conservation is avoidable. However, there is an urgent need to consider how expanding PAs and OECMs to achieve 30×30 can meet ethical aspirations for more inclusive conservation. This is especially pertinent for IPLCs, who are most directly affected by conservation policies and programs but whose voices are seldom accounted for in global decisions (Martinelli & Martinelli, 2024). Respecting IPLCs’ perspectives and incorporating them meaningfully into decisions on expanding PAs will help ensure national and international conservation efforts are equitable and effective and do not perpetuate historical injustices.
